# Supplementary material for: Association between oral microbiome and seven types of cancers in East Asian population: a two-sample Mendelian randomization analysis
Source: Front Mol Biosci. 2023 Nov 21;10:1327893. doi: 10.3389/fmolb.2023.1327893 (PMC10702768; doi:10.3389/fmolb.2023.1327893)
Supplement: Supplementary file 1 [file DataSheet1.ZIP › Supplementary Materials/MR plots for tongue/tongue═╝/Ovarian cancer/pheno.3249_to_ovarian cancer_forest.pdf]

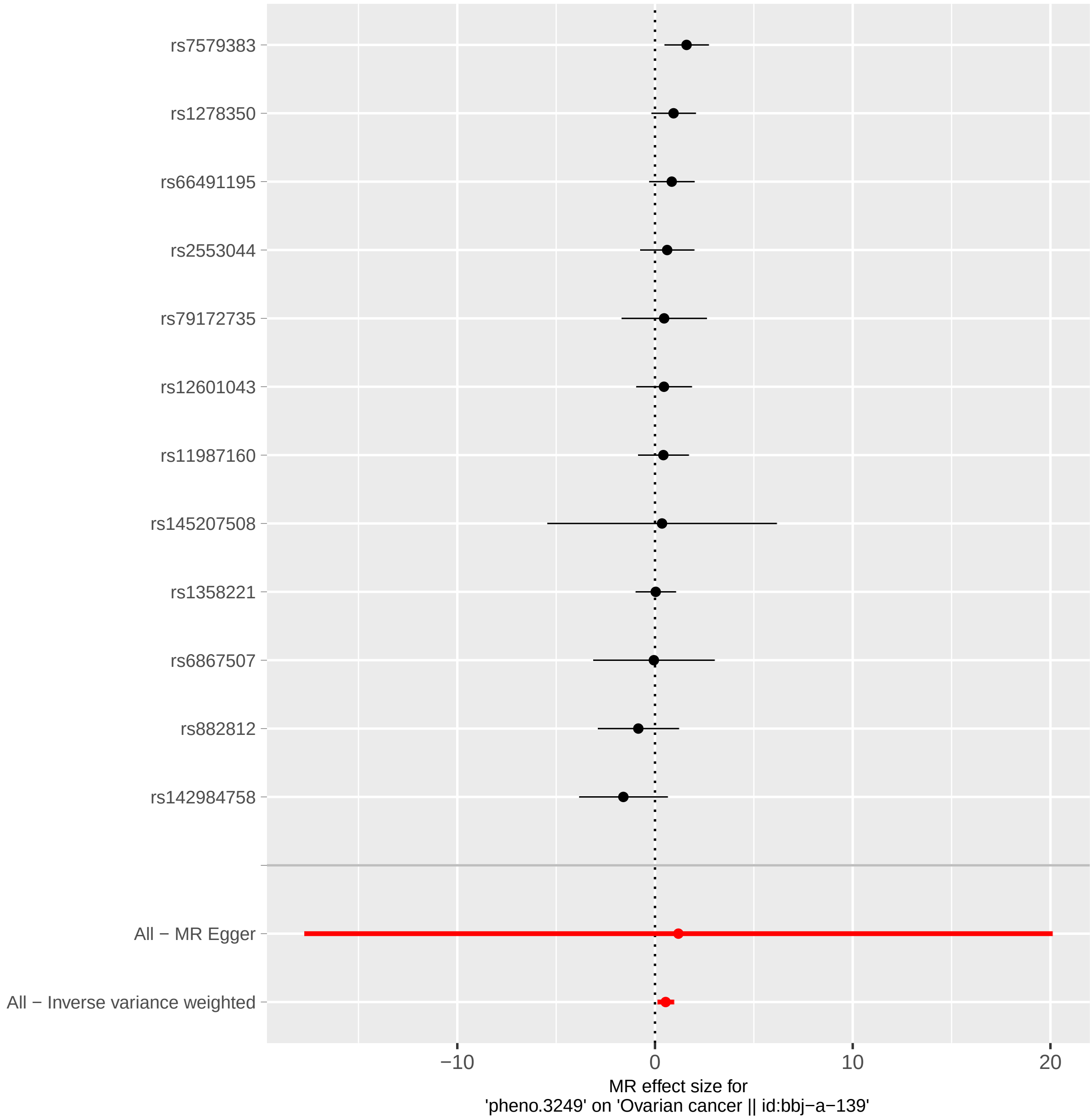
rs7579383
rs1278350
rs66491195
rs2553044
rs79172735
rs12601043
rs11987160
rs145207508
rs1358221
rs6867507
rs882812
rs142984758
All − MR Egger
All − Inverse variance weighted
−10
0
10
20
MR effect size for
'pheno.3249' on 'Ovarian cancer || id:bbj−a−139'
